# *N_e_* Does Not Provide Sufficient Information on Allelic Variation: Suggestions to Fill the Gap

**DOI:** 10.1111/eva.70219

**Published:** 2026-03-20

**Authors:** Sean M. Hoban, Brenna R. Forester, Roberta Gargiulo, Austin C. Koontz, Alicia Mastretta‐Yanes, Joachim Mergeay, Ivan Paz‐Vinas, Coalition for Conservation Genetics, Linda Laikre

**Affiliations:** ^1^ Center for Tree Science Morton Arboretum Lisle Illinois USA; ^2^ The Committee on Evolutionary Biology The University of Chicago Chicago Illinois USA; ^3^ U.S. Fish and Wildlife Service Raleigh North Carolina USA; ^4^ Royal Botanic Gardens, Kew Richmond UK; ^5^ Research Institute for Nature and Forest Geraardsbergen Belgium; ^6^ Applied Population Genetics and Conservation Genomics, Department of Biology KU Leuven Leuven Belgium; ^7^ Université Claude Bernard Lyon 1, LEHNA UMR 5023, CNRS, ENTPE Villeurbanne France; ^8^ Department of Zoology Stockholm University Stockholm Sweden

## Abstract

Conservation success depends on translating theory into practical guidance and tools that are relevant and useful for non‐scientists. While the complexity of population genetics has challenged the usage of straightforward metrics for conservation, several practical guidelines have been advanced, such as those regarding effective population size (*N*_*e*_). Allendorf et al. highlight limitations of *N*_*e*_ as a metric for practical use. Specifically, they demonstrate that while *N*_*e*_ is sufficient for predicting heterozygosity, it is not predictive of the number of alleles, another key variable in conservation genetics. This has important implications for *N*_*e*_‐based metrics, such as the *N*_*e*_ 500 indicator recently adopted in the Convention on Biological Diversity's Kunming–Montreal Global Biodiversity Framework. As developers and advocates of the *N*_*e*_ 500 indicator, we agree with this assessment, and acknowledge that *N*_*e*_ does not comprehensively predict changes in allelic variation. In this article we briefly summarize several major points in Allendorf et al. and provide practical suggestions to better account for allelic variation during indicator assessments. These suggestions include reporting major declines in *N*_*c*_ as part of genetic assessments, clearly articulating the intention and caveats of the *N*_*e*_ 500 indicator, integrating simulations into genetic assessments, and assessing the number of genetically distinct populations. We conclude that the *N*_*e*_ 500 indicator remains a valuable metric uniquely capable of capturing critical aspects of a species' genetic status while remaining accessible and interpretable to policymakers and other non‐geneticists. By acknowledging the limitations of focusing solely on *N*_*e*_ and providing options for more thorough and nuanced understandings of genetic diversity, we hope to guide future usage of the *N*_*e*_ 500 indicator and help bridge the gap between conservation genetics theory and practice.

“The possible loss of allelic variation should be taken into account for evaluating the conservation status of species as well as effective population size.” Allendorf et al. (2024).

Allendorf et al. (2024) recently highlighted limitations of the use of effective population size (*N*_*e*_) for assessing the genetic diversity of populations, namely that *N*_*e*_ does not fully portray the genetic vulnerability of species' populations. This point has important policy implications. In 2020, Laikre, Hoban, and colleagues developed several simple indicators of species' genetic status, grounded in principles of conservation biology and genetics (Laikre et al. 2020; Hoban et al. 2020), including “the proportion of populations maintained within species” and “the proportion of populations within species with an effective size greater than 500” (termed the “*N*_*e*_ 500 indicator”). An *N*_*e*_ of 500 should maintain heterozygosity and quantitative genetic variation underlying traits within populations (Franklin and Frankham 1998). A practical framework was also developed for reporting these indicators in the Convention on Biological Diversity (CBD) Kunming–Montreal Global Biodiversity Framework (KMGBF; CBD 2022), especially Target 4, “…to maintain and restore… genetic diversity within and between populations… to maintain adaptive capacity” (Laikre et al. 2020; Hoban et al. 2020). In December 2022, these and other genetic indicators were included in the KMGBF. Parties to the CBD will strive to measure them by 2026 and 2029 and make policy decisions based on the state of the indicators. Certain provincial (Jalilvand and Dileo 2025) and national (JNCC 2025) biodiversity assessments have already begun reporting the *N*_*e*_ 500 indicator for given species, and *N*_*e*_ is referenced in other biodiversity policies, such as guidance documents for the EU Habitats Directive (Council of the European Communities 1992).

We agree with Allendorf et al. (2024), and provide this commentary to summarize their messages and offer five practical suggestions regarding assessments of populations' genetic composition in the context of the KMGBF. We conclude that, like all indicators (e.g., the Red List Index, the percent of land and water in protected areas, atmospheric CO_2_ concentration, etc.), the *N*_*e*_ 500 indicator has simplifications, requires caveats, and is not comprehensive—but nevertheless strikes a useful balance between the scientific and policy aspects of conserving genetic diversity.

## 1 Main Messages of Allendorf et al. (2024)

Allendorf et al. (2024) emphasize that “heterozygosity is a good predictor of the potential of a population to evolve in the short term… [but] the limit of response to selection over many generations is determined by the initial allelic variation present.” They explain that species' populations need to adapt “quickly” (which requires high heterozygosity) but also to adapt “well,” for example, to transition to a new adaptive peak, else the population may be extirpated in the face of changing environmental conditions. This principle is illustrated by comparing the expected response to selection in several hypothetical bird populations with different initial frequencies of alleles contributing to flight endurance. In one system, the response to selection is faster due to high heterozygosity, but ultimately the absolute fitness needed (achieved through a level of flight endurance) cannot be reached due to the absence of certain alleles from the population. In another system, the initial response to selection is slower but the necessary fitness is ultimately reached because certain alleles are present, even if initially at low frequencies. They also use several examples (namely, major histocompatibility complex loci in animals and R and S genes in plants) to emphasize that several important adaptive loci have large numbers of alleles.

After demonstrating the importance of maintaining alleles, Allendorf et al. reach their key thesis: *unlike heterozygosity, the number of alleles cannot be predicted from N*_*e*_ *alone*. This is because the number of alleles depends on both *N*_*e*_ and census size (*N*_*c*_), so populations that have the same *N*_*e*_ but different *N*_*c*_ can experience different degrees of allelic loss, indicating that the *N*_*e*_ 500 indicator can neglect a key aspect of genetic composition and adaptive capacity. Simulations and analytical predictions are used to illustrate that this is true in equilibrium and non‐equilibrium populations, and the authors provide several examples demonstrating how both population size dynamics and demographic factors impact the number of alleles. First, short intense bottlenecks cause a greater loss of alleles than long “diffuse” bottlenecks, as noted earlier by Allendorf (1986). Second, unequal numbers of males and females impact *N*_*e*_, heterozygosity, and number of alleles, as does the nonrandom production of offspring. Third, they remind us that *N*_*e*_ over multiple generations is the harmonic mean of *N*_*e*_ over those generations, as noted by Waples (2005) and others.

They close with a key practical takeaway: large losses of alleles occur when *N*_*c*_ is substantially reduced, even when the post‐reduction *N*_*c*_ remains relatively large (i.e., greater than 5,000). As noted originally by Ryman et al. (1995), declines in *N*_*c*_ from very large population sizes are of particular concern (as in some overharvested marine fish; Ryman et al. 1995). Exclusive reliance on the *N*_*e*_ 500 indicator could fail to account for genetic impacts of such massive declines in *N*_*c*_ (e.g., > 90%), since most populations could still have an *N*_*e*_ of ~500 (roughly, for many species, an *N*_*c*_ of ~5,000) or greater. Therefore, using only the *N*_*e*_ 500 indicator to assess the genetic status of populations may fail to identify potentially large losses of allelic variation and adaptive capacity within populations.

**FIGURE 1:**
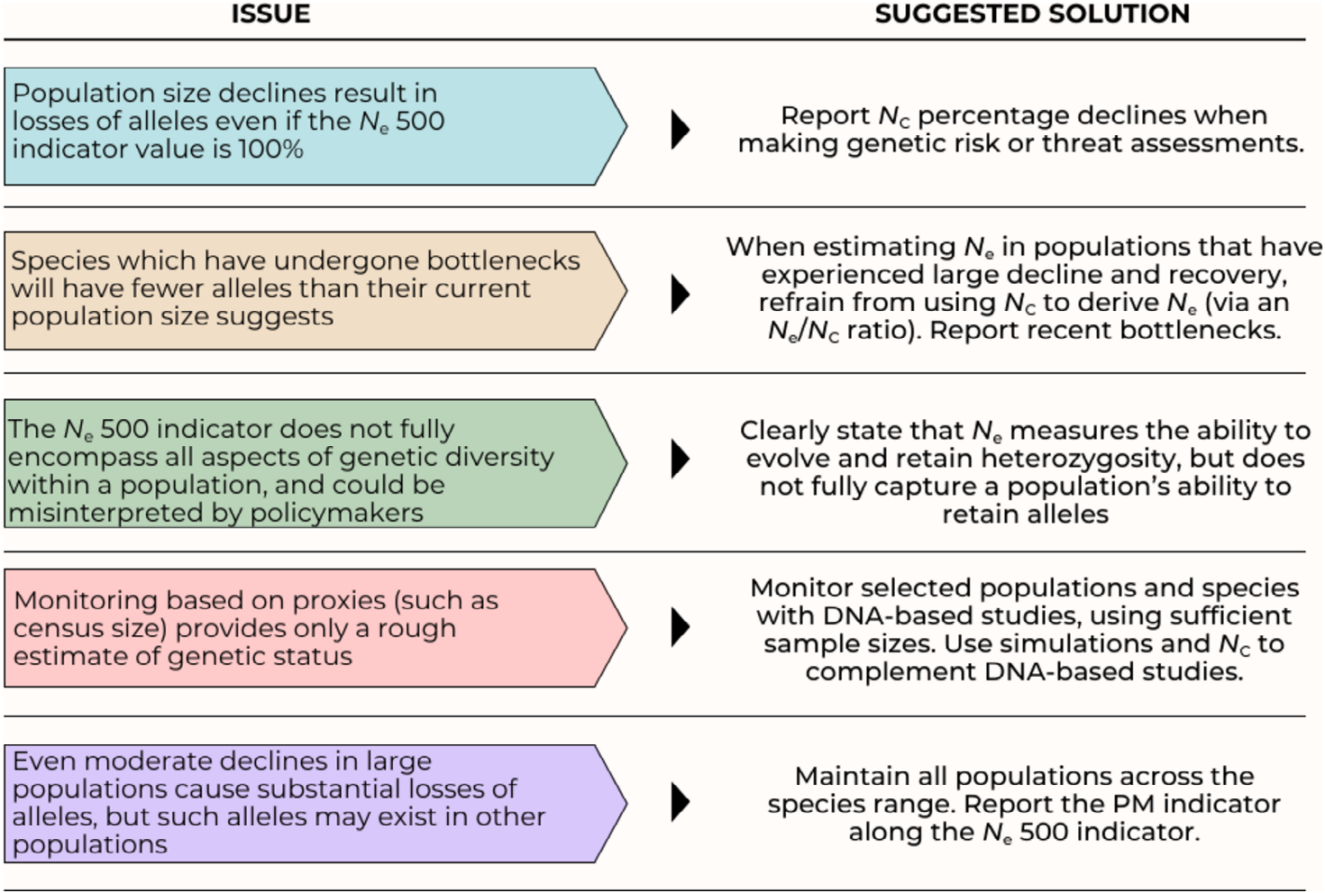
Issues raised surrounding the limitations and interpretation of the *N*_*e*_ 500 indicator, and the suggested solutions.

These points emphasize the fundamental importance of population genetic theory (also shown in Allendorf et al. 2010) and illustrate the complex ways demographic and genetic processes can interact. Allendorf et al. (2024) conclude that “there are no simple recommendations similar to the 50/500 guideline… [but] possible loss of allelic variation should be taken into account for evaluating the conservation status of species as well as effective population size”. We agree that preventing large loss of *N*_*c*_ is paramount, and that *a narrow focus on N*_*e*_ *can be problematic*; the challenge is *how* such evaluations of species conservation status can take loss of allelic variation into account. Here we make several complementary suggestions (see Figure 1), acknowledging these are all imperfect solutions.

## 2 Suggestions

### 2.1 Suggestion 1: Monitor Declines in *N_c_* as Well as *N*_*e*_ as Part of Genetic Risk Assessments

First, in the absence of genetic monitoring, we suggest that assessments of the genetic status of populations should calculate or infer the percentage of *N*_*c*_ decline, as a first indication of possible loss of alleles. The percentage decline in population *N*_*c*_ could be presented as an indicator itself, or could be translated to approximate allelic loss via the approach in figure 4 in Allendorf et al. (2024). Data on *N*_*c*_ declines could be obtained by leveraging existing monitoring programs that regularly census populations (Hoban et al. 2020). Existing tools for computing the *N*_*e*_ 500 indicator include fields to capture *N*_*c*_ for each population (Mastretta‐Yanes, Suárez, et al. 2024). Otherwise, loss of a population's habitat or area of occupancy may roughly approximate declines in *N*_*c*_, or at least carrying capacity, for populations and species where habitat loss is a primary threat (Thurfjell et al. 2022). Some approximations of *N*_*c*_ and *N*_*e*_ declines in populations of hundreds of species do exist (Ceballos et al. 2017, 2020; Mastretta‐Yanes, da Silva, et al. 2024), and imply that huge losses of allelic diversity have occurred (at least, within populations—see Suggestion 5). Even rough estimates from local knowledge holders (e.g., “has the population declined to less than half its size in recent generations?”) can be informative. Lastly, DNA‐based assessments can also approximate the magnitude of decline (Luikart et al. 2010; Antao et al. 2011).

A multi‐faceted, policy‐relevant Genetic Risk Index has already been proposed which integrates metrics related to genetic vulnerability, including past *N*_*c*_ declines, with current and future adaptive capacity (Kriesner et al. 2020). Systematic monitoring of declines in population‐level *N*_*c*_ is also relevant for demographic and ecosystem functioning (Lande 1988; Spielman et al. 2004). Thus, multi‐faceted indicators could help connect these levels of biodiversity.

However, the question arises: what magnitude and duration of *N*_*c*_ decline should signal alarm for the conservation of genetic diversity within a population? One answer could be found in species that have successfully survived bottlenecks; however, populations observed today, including those which have experienced severe losses of alleles (e.g., the two flowering plants mentioned by Allendorf et al. 2024), reflect survivorship bias, because we cannot know how many populations (or species) failed to survive. Alternatively, simulations of bottleneck events in populations with realistic genetic architectures, inbreeding effects, and life‐history traits have the potential to help inform how different bottleneck parameters (i.e., magnitude and duration) would impact a given population's or species' viability as a function of both demographic and genetic factors. Simulations like these require large amounts of data to parameterize, however (e.g., Kardos et al. 2023; Robinson et al. 2022), and have substantial caveats in their interpretation (Kardos et al. 2024). Due to these limitations, these approaches will likely remain limited in their application and value for most at‐risk species.

### 2.2 Suggestion 2: Use Caution When Deriving *N_e_* Based on *N*_*c*_ After Severe Bottlenecks Have Occurred

Second, considering the importance of bottlenecks to allelic variation and the impact the harmonic mean of recent *N*_*e*_ has on the current *N*_*e*_, *the use of current N*_*c*_ *as a way to derive N*_*e*_ (*using an N*_*e*_ */N*_*c*_ *ratio*) *should be considered inappropriate for species or populations which have experienced severe bottlenecks with subsequent recovery in N*_*c*_, especially in species with long generation times (e.g., Stoffel et al. 2018). Severe bottlenecks will greatly reduce the number of alleles in a population, and furthermore, shifts in allele frequencies and intensified genetic drift can lead to secondary losses even after a bottleneck, especially of alleles which were rare pre‐bottleneck (Nei et al. 1975; Cornuet and Luikart 1996). This stipulation on when to use the *N*_*e*_ 500 indicator is straightforward if good records exist on recent fluctuations in *N*_*c*_, which emphasizes the importance of systematic calculation of census sizes for species of conservation concern.

Fortunately, current guidance on usage of the *N*_*e*_ 500 indicator explicitly highlights such situations where the *N*_*e*_/*N*_*c*_ ratio conversion should not be used (Mastretta‐Yanes, Suárez, et al. 2024). In such cases, current and historical effective population size estimates based directly on genetic data should be obtained, and complemented by knowledge on current and historic *N*_*c*_. Additionally, emphasis should be put on maintaining as many unique populations as possible (see Suggestion 5).

### 2.3 Suggestion 3: Accurately Describe What the *N*_*e*_ Indicator Does and Does Not Reflect

Third, it is important to describe precisely what the *N*_*e*_ 500 indicator is meant to convey, especially when explaining it to conservationists and policymakers. *The N*_*e*_ *500 indicator should be understood as an indicator of the maintenance of adaptive potential going forward*, or the prevention of further erosion. Being based on contemporary *N*_*e*_, it measures if a population is large enough to maintain the heterozygosity it has now and the population's short‐term evolvability. Additionally, since heterozygosity is proportional to the additive genetic variation within a population, *N*_*e*_ tracks the adaptive potential of quantitative traits. Effective population size is *not* a measure of how much adaptive potential is retained relative to the past. Two populations might have the same current *N*_*e*_ and heterozygosity and therefore similar abilities to respond to selection in the near term, but may nevertheless have retained different numbers of alleles at individual loci. Target 4 of the KMGBF focuses on *maintaining* existing levels of genetic diversity, and given the immediacy of the biodiversity crisis, an indicator of current adaptive capacity like the *N*_*e*_ 500 indicator is well‐suited for that goal (though *restoring* genetic diversity remains challenging if large amounts of allelic diversity are lost; see Suggestion 5).

We also emphasize that the *N*_*e*_ 500 indicator is not meant to reflect the complete genetic status of a species. Instead, it is meant to guide prioritization of genetic conservation efforts, offer an overall signal of the genetic status of species at a national level, track progress over time, and identify priority populations needing further study from a genetic standpoint (Hoban et al. 2024). Comprehensive assessment of genetic status would include other processes known to affect genetic diversity (e.g., lack of recruitment and habitat fragmentation; Hollingsworth et al. 2020; Kriesner et al. 2020).

### 2.4 Suggestion 4: When Feasible, Complement the *N*_*e*_ 500 Indicator With DNA‐Based Monitoring for Selected Species (but Use Large Sample Sizes)

Fourth, when feasible, government agencies and NGOs should monitor genetic diversity directly using DNA‐based approaches (facilitated by the ever decreasing cost of DNA sequencing) to detect allele loss, such as in Sweden (Andersson et al. 2022; Dussex et al. 2023; Kurland et al. 2023; Saha et al. 2024). As stated above, genetic indicators like *N*_*e*_ 500 are useful and practical, but do not reflect the status of the whole genetic composition of populations (Hoban et al. 2024). Monitoring allelic losses in carefully selected species may, for instance, allow inference of approximate losses in populations of species that are closely related or have similar biological characteristics (Gargiulo et al. 2025), although for most countries globally, this remains a distant goal.

DNA‐based monitoring must be mindful of required sample sizes (Meirmans 2015). In Section 6.4, “Genetic monitoring,” Allendorf et al. (2024) state that DNA‐based monitoring of the number of alleles requires large sample sizes. This implies that *sample sizes of a few tens of individuals often used in DNA studies are often not sufficient to monitor allelic diversity loss*. Small sample sizes will exclude rare alleles, making tracking the loss or maintenance of those alleles impossible; therefore, large sample sizes, even on the order of magnitude of the actual *N*_*c*_, are necessary for truly estimating the loss of rare or private alleles (Lockwood et al. 2007; Koontz et al. 2026). An important corollary of this is that *past estimates of the magnitude of loss of alleles may be dramatically underestimated* (e.g., Leigh et al. 2019; Pinsky and Palumbi 2014).

For species where sufficient sampling for monitoring is not possible, we emphasize that simulations (see Allendorf et al. 2024, figure 4) based on the observed changes in *N*_*c*_ (which can be directly monitored; see Suggestion 1), and species biological characteristics (such as fecundity and variation in reproductive output) can be used to infer allele losses that have occurred and those likely to occur in the future (Kurland et al. 2023; Gargiulo et al. 2025). Software for population genetic simulations has existed for several decades, is affordable and fast, and is an opportunity for managers and geneticists to collaborate to understand and interpret genetic losses (Hoban et al. 2012; van Wyk et al. 2017).

### 2.5 Suggestion 5: Maintain Populations Across Species' Ranges

Our fifth suggestion is to ensure allelic variation is maintained within a species by conserving genetically distinct populations of that species. While Allendorf et al. (2024) focus on a single populations' *N*_*e*_, the frequency of each allele often differs among populations in real multi‐population systems. For instance, an allele could occur at low frequency in some populations and high frequency in other populations, as shown in many studies across environmental clines or along a front of expanding populations. Some alleles might be private to certain populations, making those populations unique and potentially beneficial (Kalinowski 2004; Szpiech et al. 2008; Paz‐Vinas et al. 2018). As such, conserving populations across the species range (even if their size is reduced) is another way of preserving allelic diversity at the species level. This is the motivation behind the proportion of populations maintained (PM) indicator, included as a complementary indicator for Goal A in the monitoring framework of the CBD KMGBF (Robuchon et al. 2023; Hoban et al. 2023). We note that maintaining genetic diversity among populations can, in some cases, help “restore” genetic diversity (also a part of KMGBF Target 4), via translocations or gene flow (an important consideration for the European Nature Restoration Regulation; O'Brien et al. 2025). Thus, ideally, all currently existing populations should be maintained.

## 3 Advantages of the *N*_*e*_ 500 Indicator

Working with managers and policymakers requires a balance between scientific accuracy and practical implementation (Frankham 2022). Although it is not comprehensive, the *N*_*e*_ 500 indicator has several advantages:

- It reflects a major aspect of genetic status, which is the ability of a population to maintain the heterozygosity and additive genetic variance necessary for adaptation.
- It is grounded in population genetics theory and empirical evidence.
- It can be estimated from DNA‐based data or, by working carefully under certain assumptions, from proxies (i.e., census sizes of populations).
- It helps communicate a target that managers should aim for, and allows for multi‐taxa (and even country‐level) summaries.
- It helps non‐geneticists to engage and learn more about genetic diversity, and to visualize genetic processes (which often are a mystery to non‐geneticists).
- It emphasizes the importance of thinking about biodiversity at the population level (as opposed to the whole species level), which is a shift for many conservationists.
- It allowed for the inclusion of “maintaining populations' adaptive capacity” as a target within the CBD's KMGBF, thereby linking the conservation and monitoring of genetic diversity to broader conservation actions.

We conclude by agreeing with Allendorf et al. (2024) that the assessment of allelic losses is vital, although direct genetic assessments of allelic losses are unavailable and/or complex for most species globally. Future work on this topic should include developing easy‐to‐use simulations for inferring losses in allelic variation and determining thresholds for signaling alarm, while advancing holistic genetic assessments that include Essential Biodiversity Variables (Hoban et al. 2021), as well as other factors (hybridization, recruitment) in a way that is accessible and interpretable to policymakers (Kriesner et al. 2020; Jeon et al. 2024). Although imperfect, the *N*_*e*_ 500 indicator remains useful and integrates genetic diversity into conservation policy and discussions, and we hope continued dialog around its usage and interpretation can guide future conservation efforts.

## Conflicts of Interest

The authors declare no conflicts of interest.

## Data Availability

Data sharing not applicable to this article as no datasets were generated or analysed during the current study.
